# Not Just Corticosterone: Further Characterization of the Endocrine Response of Kemp’s Ridley Sea Turtles (*Lepidochelys kempii*) Reveals Elevated Plasma Aldosterone Concentrations During Field Capture Events

**DOI:** 10.3390/ani15040600

**Published:** 2025-02-19

**Authors:** Charles J. Innis, Katherine M. Graham, Cody R. Mott, Kristen M. Hart, David Roche, Michael S. Cherkiss, Elizabeth A. Burgess

**Affiliations:** 1 Anderson Cabot Center for Ocean Life, New England Aquarium, Boston, MA 02110, USA; katie.m.graham320@gmail.com (K.M.G.); eburgess@neaq.org (E.A.B.); 2 Inwater Research Group, Inc., Jensen Beach, FL 34957, USA; cmott@inwater.org; 3 Wetland and Aquatic Research Center, U.S. Geological Survey, Davie, FL 33314, USA; kristen_hart@usgs.gov (K.M.H.); droche@usgs.gov (D.R.); mcherkiss@usgs.gov (M.S.C.)

**Keywords:** Kemp’s ridley sea turtle, *Lepidochelys kempii*, hormone, adrenal, aldosterone, corticosterone, thyroxine, thyroid, capture, trawl

## Abstract

To develop safe and effective management policies, it is important to understand the physiologic effects of fishing interactions and scientific research methods on endangered marine species. In the present study, validated assays for plasma corticosterone, free thyroxine (fT4), and aldosterone were used to assess the endocrine status of 61 presumed healthy, wild Kemp’s ridley sea turtles (*Lepidochelys kempii*) that were captured for separate ecological studies using two capture methods (trawl net n = 40; manual capture n = 21). Plasma hormone concentrations were also assessed in relation to eight clinical plasma biochemical analytes. Corticosterone and aldosterone concentrations were moderately high after capture, with significantly higher concentrations in turtles captured by trawl net vs. manual capture. Free thyroxine concentrations were within previously published ranges for healthy individuals of this species. Clinical biochemical data revealed moderately elevated potassium and lactate concentrations in many individuals, with significantly greater lactate concentrations in trawl-captured turtles. Aldosterone concentrations were positively correlated with corticosterone. The results of the present study indicate that Kemp’s ridley sea turtles have robust adrenocortical activity immediately after capture, resulting in high plasma concentrations of corticosterone and aldosterone. Researchers who use such methods to access sea turtles can consider these results in planning careful and efficient field studies.

## Simple Summary

To develop safe management policies, it is important to understand the effects of fishing interactions and scientific research on endangered marine species. In this study, concentrations of three important hormones, corticosterone, thyroid hormone, and aldosterone, were determined for 61 healthy, wild Kemp’s ridley sea turtles (Lepidochelys kempii) that were captured for separate scientific studies using two capture methods (a trawl net towed by a boat, 40 turtles; or capture by hand or a hand-held net, 21 turtles). Hormone concentrations were assessed in relation to eight other blood parameters, such as sodium and blood sugar. Corticosterone and aldosterone concentrations were moderately high after capture, with significantly higher concentrations in turtles captured by trawl net vs. manual capture, indicating a substantial stress response. Thyroid hormone concentrations were within previously published ranges for healthy individuals of this species. Other blood data revealed evidence of physical exertion, with some markers of exertion being significantly greater in trawl-captured turtles. The results of this study indicate that captured Kemp’s ridley sea turtles have a robust hormonal stress response, resulting in high plasma concentrations of corticosterone and aldosterone. Researchers who use such methods to access sea turtles can consider these results in planning careful and efficient field studies.

## 1. Introduction

Sea turtles have been negatively impacted by habitat loss, unsustainable hunting and the consumption of eggs, vessel collisions, fishery interactions, pollution, disease, and severe weather events [1,2,3,4,5,6,7,8]. Kemp’s ridley sea turtle (*Lepidochelys kempii*) is critically endangered [8], having suffered a 99% reduction in its nesting population between the 1940s and 1980s [9]. Adults of this species typically inhabit the Gulf of Mexico and females nest primarily on beaches in Mexico and Texas. Binational conservation efforts have been moderately successful, but the nesting population continues to fluctuate, remaining well below historical estimates [9].

To inform conservation activities, biologists study the ecology of sea turtles using a variety of methods. Under appropriate legal authorizations, field capture techniques are used to access sea turtles to evaluate their health, response to in-water stressors (e.g., fishery interactions and dredging operations), demographics, and habitat use [1,10,11,12,13,14,15,16,17]. While generally considered safe, capture inevitably results in some degree of physiologic stress, which can be partially characterized by blood analysis [1,10,11,12,13,14,15,16].

Hormones secreted by the hypothalamus (corticotropin-releasing hormone, CRH), pituitary (adrenocorticotropic hormone, ACTH), and adrenal glands (mineralocorticoids and glucocorticoids) regulate the vertebrate hypothalamic–pituitary–adrenal axis (HPAA). Mineralocorticoids, such as aldosterone, regulate blood volume, plasma electrolyte concentrations, and blood pressure (e.g., the renin–angiotensin–aldosterone system) [18]. Aldosterone is found throughout vertebrate species, including all orders of reptiles [5,19,20,21,22,23,24,25,26,27,28]. Most studies of aldosterone in reptiles have been conducted in laboratory settings, with only two studies documenting aldosterone concentrations from free-ranging reptiles, including American alligators (*Alligator mississippiensis*) and leatherback sea turtles (*Dermochelys coriacea*) [26,27]. Although aldosterone is likely to be very important for turtles that live in the sea, it has not been extensively studied [5,27,28].

Plasma concentrations of glucocorticoids often increase in response to stressors, facilitating energy availability (e.g., gluconeogenesis and selective cellular glucose utilization) and altering behavior. While beneficial for the short-term response to stressors, elevated glucocorticoid concentrations inhibit growth, reproduction, and the immune response, which may be adaptive in the short-term but is deleterious if prolonged [29]. The glucocorticoid corticosterone is the most commonly characterized “stress hormone” for turtles. Elevated concentrations are seen in turtles affected by various fishery interactions, disease states, capture methods, long-distance transportation, and injuries [11,30,31,32,33,34,35,36]. Studies of several marine mammal species, one study of Kemp’s ridley sea turtles, and one study of leatherback sea turtles indicate that aldosterone concentrations often correlate positively with glucocorticoid concentrations and are increased in the presence of stressors [5,27,37,38].

Thyroid hormones affect seasonal transitions, energy availability during exercise, and the metabolic rate [39]. Several forms of thyroid hormone have been evaluated in sea turtles [5,14,33,34,40,41,42,43,44]. Concentrations of free thyroxine (fT4) are suppressed in cold-stunned Kemp’s ridley sea turtles but elevated in entangled, stranded, and nesting leatherback sea turtles [14,27,33]. Free thyroxine concentrations in healthy, captured leatherback turtles (for ecologic investigations) are relatively low (14,27].

The clinical plasma biochemical analytes of sea turtles (e.g., glucose, electrolytes, lactate, and urea) are well-studied, informative, and often show expected correlations with endocrine analytes [1,5,14,32,33,34]. For example, the modest elevation of plasma corticosterone that is seen after long-distance vehicle transport of Kemp’s ridley sea turtles is correlated with a modest elevation of blood glucose concentrations [34].

In the present study, we utilized archived, frozen plasma samples to retrospectively assess the adrenal and thyroid status of Kemp’s ridley sea turtles captured in the field using one of two methods. We hypothesized that plasma concentrations of aldosterone, corticosterone, and fT4 would be elevated relative to previously published data for captive, acclimated conspecifics [5]. We also hypothesized that concentrations of these hormones would be greater in turtles captured by mechanical methods (trawler) than in turtles captured by manual methods. Finally, we hypothesized that corticosterone concentrations would positively correlate with plasma glucose concentrations and that aldosterone concentrations would correlate positively with plasma sodium and negatively with plasma potassium concentrations.

## 2. Materials and Methods

### 2.1. Study Design

This retrospective study utilized frozen archived plasma samples to assess the physiologic status of Kemp’s ridley sea turtles that had been previously captured and sampled for ecologic studies by one of two methods, as described below. As such, we opportunistically used all available samples; thus, a specific a priori sample size was not assigned. Samples had been collected from healthy individuals based on external examinations; thus, no samples were excluded from this study. No animals were subjected to painful procedures; therefore, analgesic methods and humane endpoints were not established for this study.

### 2.2. Study Animals and Blood Sample Collection

#### 2.2.1. Trawl Net Capture and Sampling

Kemp’s ridley turtles were captured by trawl nets as previously described [17] during research or mitigation events (relocation of turtles from dredging sites) in the Gulf of Mexico, USA from 18 May–22 June 2016 (n = 10, Caminada II Borrow Site, LA, USA), 7 September–21 October 2018 (n = 22, Pascagoula, MS, USA), and 7–16 May 2019 (n = 8, Ship Shoal, LA, USA). Research trawling involved a contracted trawler operating 12 h a day in areas predetermined by U.S. Geological Survey (USGS) staff. Turtles captured during research trawls were released at the capture site, while those captured during mitigation trawling were released offshore, away from dredge sites. To limit the underwater confinement time of turtles within trawls, net drag times were limited to 30 min and conducted at speeds of 3 to 6 km/h.

Turtles were examined, measured, and weighed. Approximately 2 mL of blood was collected from the external jugular vein using 21-gauge BD Vacutainer blood collection needles and lithium heparin tubes (Becton, Dickinson and Company, Franklin Lakes, NJ, USA). Although the time elapsed between capture and sample collection was not specifically recorded, it was approximately 45 min during research expeditions. During mitigation expeditions, sampling time was variable and of greater duration (but not recorded) because USGS biologists had to be transported to the trawlers via secondary vessels when turtles were captured. Samples were refrigerated or kept chilled in a cooler until centrifugation to harvest plasma. The time between blood collection and centrifugation varied depending on field location but was between 1 and 12 h. Plasma samples from 2016 were initially stored at −20 °C and later stored (2018) at −80 °C until analysis. Samples from 2018 and 2019 were stored at −80 °C until analysis.

#### 2.2.2. Manual Capture and Sampling

Kemp’s ridley sea turtles were hand-captured or dip-net-captured in the Big Bend region of Florida, USA from 10–18 May 2018 (n = 9), 26–28 September 2019 (n = 5), and 27–30 August 2020 (n = 7) using previously described methods [45]. Within one hour of capture (exact time not recorded), the external jugular venipuncture site was disinfected with iodine and alcohol, and approximately 3 mL of blood was collected using 20- or 22-gauge needles and lithium heparin vacuum tubes. Following blood collection, turtles were examined, measured, and weighed. On completion of examination and sampling, each turtle was identified on the carapace with a temporary livestock marker to avoid repeated capture. Blood samples were chilled in a cooler until centrifuged to harvest plasma. The duration between blood collection and centrifugation varied but was between 2 and 12 h. Samples were stored at −80 °C until analysis.

### 2.3. Plasma Analyses

Radioimmunoassay kits were used to quantify corticosterone (corticosterone double antibody ^125^I RIA kit, catalog #07-120103, MP Biomedicals, Solon, OH, USA) and fT4 (free T4 coated tube RIA kit, catalog #06B-257214, MP Biomedicals, Solon, OH, USA), and an enzyme immunoassay kit was used to quantify aldosterone (ALD: DetectX^®^ aldosterone kit, catalog #K052, Arbor Assays, Ann Arbor, MI, USA). These commercially available kits had been previously validated for Kemp’s ridley sea turtle plasma [5,34]. Assay methods were performed according to the manufacturer’s instructions, except that an additional low standard was included in each standard curve to increase the detection range for corticosterone (0.0625–5 ng/mL, 7 standards), fT4 (1.5–120 pg/mL, 6 standards), and aldosterone (0.98–4000 pg/mL, 7 standards). Additionally, the corticosterone assay was performed at 50% volume (i.e., all reagent volumes were reduced to 50% of that stated in the manufacturer’s protocol) to minimize the volume required from each sample; and the aldosterone assay used the manufacturer’s overnight protocol (i.e., incubating overnight at 4 °C). To prepare for aldosterone measurement, plasma samples were extracted with ethyl acetate solvent prior to the assay. Ethyl acetate (250 µL) was added to plasma (250 µL) in a glass tube and then gently vortexed for 30 s. Layers were allowed to separate, and the top organic layer was removed and placed into a clean glass tube. These extraction steps were repeated twice more, pooling the ethyl acetate supernatant layers. The resulting final supernatant was dried by evaporation under compressed air. The dried product was reconstituted in 250 µL of assay buffer (catalog #X065, Arbor Assays) for aldosterone analysis.

All controls, standards, and samples were assayed twice. Assays were repeated for samples with a coefficient of variation between replicates >10%. Quality control (i.e., precision and reproducibility) was monitored by measuring the concentration of low (~70% binding) and high (~30% binding) control samples. Inter-assay coefficients of variation were aldosterone: 11% and 6% (n = 7 assays); corticosterone: 7% and 5% (n = 5 assays); and fT4: 3% and 3% (n = 4 assays) for high and low controls, respectively. Final plasma concentrations for fT4 and aldosterone data were reported as pg/mL of the immunoreactive hormone, while corticosterone concentrations were reported as ng/mL of the immunoreactive hormone.

After acquiring hormone data, if the remaining plasma volume allowed, biochemical data were generated using the Stat Profile Prime Plus VET Critical Care Analyzer (NOVA Biomedical, Waltham, MA, USA), including sodium, potassium, chloride, ionized calcium (iCa), ionized magnesium (iMg), glucose, lactate, blood urea nitrogen (BUN), and the sodium/potassium ratio (calculated).

### 2.4. Statistical Analysis

Hormone data for aldosterone and corticosterone were transformed (log_10_) to adjust for a skewed, non-normal distribution. Levene tests were conducted to confirm the homogeneity of variances. To determine whether turtles captured by the two different methods exhibited significant differences in plasma hormone and biochemical analyte concentrations, we conducted a multivariate ANOVA, with straight carapace length (SCL notch-to-tip) included as a covariate. We performed Spearman’s rank–order correlations to evaluate the relationships between plasma hormones and biochemical analytes for each capture method group. This non-parametric test, which employed untransformed data for all variables, was chosen for its robustness to outliers and its capability to detect both linear and non-linear monotonic relationships. Turtles were conservatively categorized as unknown sex if their straight carapace length was <60 cm [46]. For turtles with an SCL ≥ 60 cm, sex was assigned based on a visual assessment of external sexual dimorphism that had been recorded at the time of examination (a much larger, longer tail with a more distal vent in males) [46]. All results are presented as mean ± standard deviation, along with the median and range of minimum and maximum values. Differences were considered significant at *p* < 0.05. All statistical analyses were performed using the statistical program IBM SPSS Statistics for Mac OS, version 28 (IBM Corp., Armonk, NY, USA). No data were excluded from the analysis.

## 3. Results

Trawl-captured turtles ranged in body weight from 17 to 36 kg (mean 26.3, median 26.5) and had an SCL of 49.1 to 67.5 cm (mean 59.9 and median 59.8), including sixteen females, four males, and twenty of unknown sex (Table S1). Manually captured turtles ranged in body weight from 3.4 to 27.4 kg (mean 17.2, median 17.3) and had an SCL of 27.9 to 57.3 cm (mean 48.4, median 49.2), all of which were of unknown sex (Table S1). SCL was tested as a covariate in the ANOVA model, but the results were non-significant (*p* > 0.05 for all analytes), indicating that differences in turtle size did not substantially account for the observed variation in the plasma hormone and biochemical analyte concentrations.

Summary hormone data are provided in Table 1 and data for individual turtles are provided in Table S1. Free thyroxine was not determined for two manually captured individuals due to insufficient plasma volume. Aldosterone (*F*_1,53_ = 34.38, *p* < 0.001) and corticosterone concentrations (*F*_1,53_ = 39.77, *p* < 0.001) were significantly higher in trawl-captured turtles than manually captured turtles. There was no difference in fT4 concentrations between the two groups. Correlation data among hormones are provided in Table 2. Corticosterone and aldosterone were positively correlated with each other for both capture scenarios, but neither was correlated with fT4 (Table 2).

Summary statistics for plasma biochemical data are provided in Table 1, and data for individual turtles are provided in Table S1. Biochemical data could not be determined for three individuals due to insufficient plasma volume (manual capture, n = 2; trawl capture, n = 1). Lactate (*F*_1,53_ = 7.70, *p* = 0.008) and iCa (*F*_1,53_ = 16.43, *p* < 0.001) were significantly greater, and chloride (*F*_1,53_ = 8.71, *p* = 0.005) was significantly lower in trawl-captured turtles. Correlations among plasma biochemical and endocrine data are provided in Table 2. Notably, among these correlations, aldosterone and corticosterone positively correlated with glucose for manually captured turtles, and corticosterone and fT4 positively correlated with glucose for trawl-captured turtles. Aldosterone and corticosterone positively correlated with potassium and BUN for trawl-captured turtles.

## 4. Discussion

Our study evaluated selected endocrine and clinical biochemical analytes for free-ranging Kemp’s ridley sea turtles that were captured by two methods. While many of these analytes have been previously studied for this species during such events, the present study is the first to evaluate plasma aldosterone and fT4. The results of our study add to the limited data on plasma aldosterone and fT4 for sea turtles and reptiles in general.

Baseline plasma aldosterone concentrations vary moderately within vertebrate species. For example, reference intervals generally range from the tens to low hundreds of pg/mL in both humans and dogs [47,48,49,50]. Aldosterone concentrations for Kemp’s sea ridley turtles in our study were at the high end of (manual capture) or much higher than (trawl capture) maximal values within these mammalian reference intervals. Mean concentrations seen in trawl-captured turtles were also higher than most previously published data for reptiles [21,23,25], including limited data for Kemp’s ridley sea turtles [5,28]. Only concentrations from Kemp’s ridley sea turtles rescued during an oil spill, and from the sea snake (*Hydrophis cyanocinctus*), are reported to be higher than those seen in trawl-captured turtles in the present study (sea snake concentrations are outliers among most other reptiles) [5,22]. While true baseline aldosterone concentrations have not yet been established for Kemp’s ridley sea turtles, data from rehabilitated conspecifics during convalescence show mean concentrations < 12 pg/mL [5]. Overall, we consider the aldosterone concentrations in the present study to be moderately high.

Corticosterone concentrations for Kemp’s ridley sea turtles in the current study were also moderately high. Concentrations were similar to those reported for conspecifics affected by stressors such as nesting, gill net capture, entanglement net capture, and vehicle transport of 21–26 h duration [12,15,34,51], and similar to concentrations for loggerhead sea turtles (*Caretta caretta*) captured by similar methods [11]. Data for healthy conspecifics suggest that baseline corticosterone concentrations may be ≤6 ng/mL [5,33,34,51]. Among other studies of corticosterone concentrations for this species, only concentrations of cold-stunned or oiled conspecifics are higher than those seen for trawl-captured turtles in the present study [5,33]. Cumulatively, the moderately high aldosterone and corticosterone concentrations in the present study indicate a moderate adrenocortical stress response, which is of greater magnitude during trawl capture than manual capture.

Free thyroxine concentrations did not vary between capture methods in the present study and were similar to those previously reported for this species under a variety of circumstances. Therefore, they are likely within the “normal” range for healthy individuals [5,33]. To date, only cold stunning appears to substantially affect fT4 concentrations for this species, with very low concentrations upon admission to the hospital [33].

Biochemical data for Kemp’s ridley sea turtles under various capture scenarios have been described [5,10,12,13,15,16]. The present study was not primarily intended to provide a general biochemical evaluation, but rather to allow for comparisons of endocrine and biochemical data. In comparison to previously published data, the biochemical results in our study were generally within expected ranges for healthy individuals of this species. However, results for some analytes included atypically high and/or low values, including potassium, lactate, glucose, and iCa. Although they were not significantly different between capture methods, potassium concentrations in our study trended somewhat higher than typically reported for this species [16,52,53], and the highest concentrations were most similar to concentrations described in previous studies of gill net capture or experimental trawling [10,15]. Elevated potassium concentrations are most likely related to exertion and/or ionic compensation for acidosis, although artifactual effects cannot be ruled out (e.g., hemolysis). Elevated potassium concentrations are notable because hyperkalemia often carries a poor prognosis in hospitalized Kemp’s ridley sea turtles [53].

Lactate concentrations were significantly greater in trawl-captured turtles than manually captured turtles in our study, but concentrations for both capture methods were relatively high. Similarly elevated lactate concentrations (e.g., >10 mmol/L) were described in previous studies of this species during a variety of capture or confinement methods [10,15], while baseline concentrations are often <2 mmol/L [10,13,53]. In our study, only six out of forty-one turtles had lactate <10 mmol/L, all of which were manually captured, while twenty turtles had lactate >19 mmol/L, all of which were trawl-captured. Collectively, observations of lactate concentrations in our study and previous studies indicate that capture induces variable degrees of metabolic acidosis, most likely the result of vigorous exertion during trawl confinement and capture [10,13,15]. Additional evidence for compensation for acidosis in our study might include the observation of significantly lower chloride concentrations in trawl-captured turtles (albeit minimally clinically relevant). Relatively lower chloride concentrations were also described by Stabenau et al. during experimental trawl exposures of Kemp’s ridley sea turtles, which was attributed to “renal compensation for extracellular acidosis” [10].

Ionized calcium concentrations were significantly greater in trawl-captured turtles in our study compared to manually captured turtles, but in general, concentrations were typical for this species [53]. It is possible that the statistical difference was due to several anomalously low results among the manually captured group (e.g., two individuals had iCa values of 0.33 and 0.35 mmol/L). Whether these low values were accurate or the result of methodological artifact cannot be determined.

Glucose concentrations did not differ between capture methods and were generally within the expected range for Kemp’s sea ridley turtles. Several individuals that were captured by trawling had the lowest or highest concentrations (i.e., glucose < 3 mmol/L, n = 2 or glucose > 8 mmol/L, n = 2), possibly related to glucose utilization during exertion or the gluconeogenic effect of elevated corticosterone concentrations, respectively.

The correlations of biochemical analytes and hormones that were detected in our study do not necessarily indicate causal mechanisms. While it is likely that certain direct effects were detected (e.g., corticosterone and glucose), it is possible that other correlations were the result of concurrent changes unrelated to direct causation. For example, elevated aldosterone concentrations are typically expected to cause hypernatremia and hypokalemia, but in the present study aldosterone was not correlated with sodium and was positively correlated with potassium for trawl-captured turtles. It is likely that such correlations are the result of concurrent physiologic changes, including high corticosteroid concentrations in response to a stressor and elevated concentrations of certain biochemical analytes during exertional acidotic conditions (e.g., potassium and lactate).

We recognize a number of limitations with our study. Because this was a retrospective study that used archived plasma samples, a detailed study design was not followed with respect to handling duration prior to blood collection, time to sample centrifugation, and sample storage. The time of blood sample collection relative to capture was not recorded, but times of 45 min or longer were typical, which certainly may have contributed to elevated corticosteroid concentrations. Samples were frozen for up to four years prior to analysis, but corticosteroid and thyroid hormones appear to be reasonably tolerant of long-term freezing and freeze–thaw cycles [54,55,56]. While manually captured turtles were generally smaller (lower SCL) than trawl-captured turtles, our analysis did not find that size affected blood analyte concentrations. Additional limitations of the retrospective design include an uneven sex ratio (and many turtles of unknown sex), variable age, weight, and geographic areas, an unequal temporal distribution of sampling, and variable sample sizes for each sampling period (i.e., sampling events were clustered in certain months in certain years). This variability prevented the statistical analysis of these factors, which is often unavoidable when using opportunistic samples from endangered free-ranging species. Based on the results of this study, prospective studies could be useful in assessing these same analytes with more attention to sampling time (i.e., collect blood as quickly as possible and record the time) and seasonal, geographic, and demographic effects.

## 5. Conclusions

Collectively, the results of our study and previous studies indicate that Kemp’s ridley sea turtles are moderately physiologically stressed during capture events, with manifestations such as elevated aldosterone and corticosterone concentrations, with somewhat more severe effects during trawl capture compared to other methods. Although many other biochemical analytes remain within the expected ranges after capture, the detected physiologic changes should continue to prompt researchers to proceed efficiently and carefully and to consider physiologic monitoring during sea turtle capture events. Our results can inform management decisions regarding the physiologic effects of trawling during fishery operations.

## Figures and Tables

**Table 1:** Summary of plasma hormone concentrations and biochemical measurements of Kemp’s ridley sea turtles captured using two different techniques: manual capture and trawler capture. Different color shades denote a significant difference in biomarker measures between groups at *p* < 0.05 (orange shades depict higher values and blue shades depict lower values). Abbreviations are fT4 = thyroxine, Na = sodium, K = potassium, Cl = chloride, iCa = ionized calcium, iMg = ionized magnesium, BUN = blood urea nitrogen, SD = standard deviation, Min = minimum, Max = maximum, and N.S. = not significant.

	Manual Capture (n = 21)					Trawl Capture (n = 40)					
	Mean	SD	Median	Min	Max	Mean	SD	Median	Min	Max	*p* Value
Aldosterone (pg/mL)	193.6	202.8	80.9	0.5	686	523.0	264.9	491.2	126	1152	<0.001
Corticosterone (ng/mL)	10.7	12.6	2.7	1.4	39	25.5	12.0	24.5	5.7	54	<0.001
fT4 (pg/mL)	1.89	0.85	1.95	0.3	3.3	2.13	0.64	2.11	1.0	3.8	N.S.
Na (mmol/L)	152	5	152	141	162	151	2	152	146	156	N.S.
K (mmol/L)	5.3	1.0	5.1	3.7	7.2	5.2	0.7	5.2	4.1	7.5	N.S.
Na:K ratio	29.8	5.0	30.3	21.5	41.3	29.3	3.5	29.1	20.4	36.9	N.S.
Cl (mmol/L)	121	4	121	110	129	119	2	119	114	124	0.005
iCa (mg/dL)	0.69	0.22	0.69	0.33	1.14	0.94	0.13	0.96	0.62	1.16	<0.001
iMg (mmol/L)	1.28	0.31	1.25	0.81	2.10	1.32	0.31	1.30	0.77	2.67	N.S.
Glucose (mmol/L)	4.6	0.7	4.3	3.4	6.1	5.2	1.5	4.8	1.8	9.2	N.S.
Lactate (mmol/L)	12.1	5.2	12.5	2.3	19.0	18.8	4.1	19.1	11.6	26.1	0.008
BUN (mmol/L)	25	7	25	10.7	37.5	21.1	7.4	21.1	9.6	40.4	N.S.

**Table 2:** Correlation matrices for plasma hormone and biochemical measurements in Kemp’s ridley sea turtles captured in the wild using (a) manual capture (n = 21) and (b) trawl capture (n = 40). Values represent calculated Spearman’s rank–order correlation coefficients (rho) as a measure of linear dependence between two variables (where rho = 1 is a totally positive correlation). Asterisks highlight positive (shaded orange) and negative (shaded blue) correlations significant at the 0.05 level (*) and 0.01 level (**). Aldo = aldosterone, Cort = corticosterone, fT4 = free thyroxine, Na = sodium, K = potassium, Cl = chloride, iCa = ionized calcium, iMg = ionized magnesium, BUN = blood urea nitrogen.

**(a) Manual Capture**												
	**Aldo**	**Cort**	**fT4**	**Na**	**K**	**Na:K Ratio**	**Cl**	**iCa**	**iMg**	**Glucose**	**Lactate**	**BUN**
Aldo	1											
Cort	0.735 **	1										
fT4	0.12	0.24	1									
Na	−0.01	0.12	0.10	1								
K	0.17	−0.14	−0.28	0.642 **	1							
Na:K ratio	−0.19	0.17	0.36	−0.540 *	−0.973 **	1						
Cl	−0.01	−0.06	0.14	0.842 **	0.643 **	−0.526 *	1					
iCa	0.20	0.42	0.12	−0.34	−0.489 *	0.529 *	−0.29	1				
iMg	0.26	0.582 **	0.07	−0.28	−0.27	0.28	−0.37	0	1			
Glucose	0.513 *	0.515 *	0.14	0.681 **	0.471 *	−0.42	0.456 *	−0.04	0.09	1		
Lactate	0.39	0.36	−0.12	0.45	0.44	−0.45	0.27	0.16	0.04	0.647 **	1	
BUN	−0.07	−0.31	0.10	0.34	0.41	−0.41	0.39	−0.457 *	−0.43	0.07	−0.08	1
**(b) Trawl capture**												
	**Aldo**	**Cort**	**fT4**	**Na**	**K**	**Na:K Ratio**	**Cl**	**iCa**	**iMg**	**Glucose**	**Lactate**	**BUN**
Aldo	1											
Cort	0.588 **	1										
fT4	0.10	0.15	1									
Na	−0.04	−0.14	0.05	1								
K	0.405 *	0.623 **	−0.06	−0.13	1							
Na:K ratio	−0.421 **	−0.654 **	0.05	0.27	−0.981 **	1						
Cl	0.11	−0.21	−0.11	0.379 *	0.03	0	1					
iCa	0.2	−0.05	0.13	0.19	0.02	0	0.2	1				
iMg	−0.05	−0.07	0.01	0.07	0.16	−0.14	0.23	0	1			
Glucose	0.19	0.442 **	0.332 *	−0.01	0.20	−0.19	−0.04	−0.17	−0.12	1		
Lactate	0.16	0.07	−0.09	0.02	0.17	−0.15	−0.11	0.332 *	−0.28	0.02	1	
BUN	0.414 **	0.374 *	−0.04	−0.14	0.31	−0.29	−0.14	0.01	−0.06	0.22	0.07	1

## Data Availability

The original contributions presented in this study are included in the article/table/Supplementary Materials. Further inquiries can be directed to the corresponding author.

## Supplementary Materials

The following supporting information can be downloaded at: https://www.mdpi.com/article/10.3390/ani15040600/s1, Table S1: Plasma hormone concentrations and biochemical measurements of Kemp’s ridley sea turtles (*Lepidochelys kempii*) captured by one of two methods. M = manual; T = trawl; SCL = notch-to-tip straight carapace length; U = unknown, F = female; M = male; Aldo = aldosterone; Cort = corticosterone; fT4 = free thyroxine; Na = sodium; K = potassium; iCa = ionized calcium; iMg = ionized magnesium; Glu = glucose; Lac = lactate, BUN = blood urea nitrogen.
