# Supplementary material for: Not Just Corticosterone: Further Characterization of the Endocrine Response of Kemp’s Ridley Sea Turtles (Lepidochelys kempii) Reveals Elevated Plasma Aldosterone Concentrations During Field Capture Events
Source: Animals (Basel). 2025 Feb 19;15(4):600. doi: 10.3390/ani15040600 (PMC11852127; doi:10.3390/ani15040600)
Supplement: Supplementary file 1 [file animals-15-00600-s001.zip › animals-3392144-SI.pdf]

Table S1: Plasma hormone concentrations and biochemical measurements of Kemp's ridley turtles (*Lepidochelys kempii*) captured by one of two methods. M=manual; T=trawl; SCL=notch-to-tip straight carapace length; U=unknown, F=female; M=male; Aldo=aldosterone; Cort=corticosterone; fT4=free thyroxine; Na=sodium; K=potassium; iCa=ionized calcium; iMg=ionized magnesium; Glu=glucose; Lac=lactate, BUN=blood urea nitrogen.

Turtle ID	Date	Capture method	SCL cm	Weight kg	Sex	Aldo pg/ml	Cort ng/ml	fT4 pg/ml	Na mmol/l	K mmol/l	Cl mmol/l	iCa mmol/l	iMg mmol/l	Glu mmol/l	Lac mmol/l	BUN mmol/l
75	5/10/18	M	51.7	19.4	U	81.15	2.71	0.30	162.0	6.91	129.1	0.66	0.92	5.11	19	29.6
406	5/10/18	M	49.2	18.3	U	45.16	1.41	1.66	151.0	5.12	125.0	0.50	1.17	4.00	7.2	24.6
416	5/11/18	M	42.7	11.3	U	167.55	2.60	1.84	151.2	6.12	121.4	0.60	1.27	4.22	10.8	32.5
422	5/11/18	M	48.1	15.8	U	11.91	2.13	3.15	161.9	5.85	128.9	0.57	1.16	5.06	17.6	26.4
424	5/12/18	M	46.2	13.6	U	36.37	1.62	1.38	155.1	6.21	125.4	0.51	1.17	4.94	11.9	33.9
401	5/12/18	M	48.4	17.0	U	353.86	2.57	2.73	155.3	7.21	126.0	0.35	0.86	4.39	8.8	37.5
413	5/12/18	M	27.9	3.4	U	80.17	5.88	2.66	148.9	4.12	117.2	0.84	1.41	4.17	12.5	25.4
418	5/13/18	M	55.0	23.6	U	57.80	1.44	0.62	149.4	6.09	116.5	0.77	1.17	4.17	18.1	23.6
426	5/13/18	M	36.4	7.2	U	80.85	2.06	1.95	140.5	3.92	110.0	0.69	0.81	4.06	7.3	21.4
140	9/26/19	M	50.4	19.1	U	29.23	2.88	1.31	156.3	5.99	120.9	0.33	1.37	5.11	13.3	20.0
467	9/26/19	M	55.1	27.4	U	21.22	2.48	2.11	154.1	4.80	122.8	0.75	1.25	3.94	12	30.4
469	9/26/19	M	53.8	22.1	U	0.51	1.73	2.55	152.4	3.69	121.3	0.67	0.94	4.17	2.3	27.9
483	9/27/19	M	54.6	23.4	U	65.45	4.68	2.33	147.0	4.18	114.2	0.70	1.71	4.00	3.5	22.9
487	9/28/19	M	47.4	13.1	U	17.57	2.39	0.35	142.7	4.58	119.1	0.89	1.48	3.44	4.3	10.7
936	8/27/20	M	47.2	16.7	U	462.24	14.67	1.68	QNS	QNS	QNS	QNS	QNS	QNS	QNS	QNS
940	8/27/20	M	46.0	17.3	U	213.15	23.53	2.25	QNS	QNS	QNS	QNS	QNS	QNS	QNS	QNS
944	8/27/20	M	57.3	21.2	U	686.14	20.26	QNS	150.9	5.06	118.5	0.54	1.57	4.89	17.4	21.8
962	8/28/20	M	49.4	16.5	U	430.61	31.30	1.51	151.4	5.00	120.5	0.71	1.33	5.28	14.4	30.7
974	8/29/20	M	45.8	14.3	U	396.90	22.95	3.27	154.9	5.09	124.8	1.13	2.10	6.06	16.3	21.8
981	8/30/20	M	50.8	19.1	U	478.44	35.41	2.28	154.8	5.08	123.4	1.14	1.33	5.83	16.5	19.6
985	8/30/20	M	53.2	21.4	U	349.63	39.09	QNS	153.9	5.00	122.9	0.84	1.25	4.33	16.2	12.1
38	9/7/18	T	59.7	NR	U	603.95	43.09	3.02	147.7	5.23	119.3	0.98	1.30	7.50	15.4	13.9
48	9/8/18	T	62.8	NR	F	284.19	17.76	2.84	149.5	4.88	114.5	0.75	1.05	3.78	19.8	21.1
56	9/8/18	T	65	NR	F	555.72	32.28	2.88	151.2	4.97	120.6	0.86	1.20	7.28	11.7	22.5
59	9/8/18	T	57.8	NR	F	585.50	30.11	2.19	151.1	5.96	119.1	1.06	1.20	5.44	25.1	26.8
68	9/8/18	T	63.2	NR	M	667.16	29.25	2.37	151.8	5.32	121.0	0.83	1.27	6.39	19.1	25.4
90	9/9/18	T	62.3	NR	F	643.81	24.21	2.11	151.1	4.79	113.6	0.78	1.20	6.72	24.8	15.4
93	9/9/18	T	65.2	NR	F	149.12	13.80	1.82	149.0	4.89	118.4	0.99	1.31	6.06	22.5	25.7
101	10/2/18	T	58.7	30.2	U	419.82	25.07	1.47	147.4	6.43	118.9	0.86	1.54	5.72	15.1	20.0
119	10/4/18	T	54.8	24.3	U	450.54	43.07	3.78	150.8	5.18	114.5	0.76	1.11	7.67	14.7	24.3
127	10/4/18	T	53	21.9	U	565.25	38.20	2.12	151.9	5.47	117.7	0.88	1.54	4.89	13.6	17.9
147	10/5/18	T	61.6	30.5	F	157.65	14.83	2.73	151.8	4.69	116.5	0.62	0.77	4.61	18.2	12.5
156	10/5/18	T	65	36.2	F	305.05	24.37	2.60	150.6	5.07	118.2	0.97	1.38	6.00	26.1	17.9
178	10/6/18	T	55.5	21.8	U	480.73	22.17	1.45	152.9	7.49	122.0	0.66	0.85	4.89	19.9	18.2

207	10/15/18	T	57.5	24.1	U	967.14	51.45	2.12	151.6	6.34	118.8	0.92	2.67	4.00	18.1	23.9
210	10/15/18	T	63	34.3	F	293.44	24.56	2.08	154.2	5.38	118.5	0.92	1.30	3.50	16.2	23.9
213	10/15/18	T	55.4	24.1	U	612.29	35.97	1.95	154.5	4.44	118.4	0.86	1.24	9.22	11.6	29.6
215	10/15/18	T	50.5	19.5	U	477.44	29.88	2.86	149.8	5.63	118.0	0.91	1.44	8.44	13.2	31.4
233	10/16/18	T	58.1	27	U	214.77	29.35	2.08	153.9	5.35	118.1	0.97	1.55	6.11	18.5	14.3
247	10/17/18	T	59.6	26.9	U	142.01	13.02	1.66	152.6	4.69	120.8	1.16	1.49	4.56	17.9	15.7
276	10/21/18	T	49.1	17	U	1151.50	23.77	2.73	149.0	5.57	118.5	1.11	1.37	4.78	17	24.6
278	10/21/18	T	53.2	20.7	U	246.17	19.65	1.28	145.7	5.53	115.8	0.91	1.86	1.83	16.1	17.5
282	10/21/18	T	62.8	30.6	F	485.30	10.96	1.65	153.2	4.94	119.5	0.95	1.62	2.83	13.7	23.2
292	5/7/19	T	59.5	28.8	U	463.03	15.74	0.98	152.2	5.25	121.7	0.96	1.23	5.11	21.6	10.0
330	5/9/19	T	51.9	22.1	U	622.05	12.56	2.14	153.7	4.96	124.2	1.08	1.47	4.28	16.4	27.9
338	5/9/19	T	62.5	NR	F	656.13	10.87	2.89	152.7	4.14	120.4	1.04	1.30	4.39	16.4	17.9
380	5/13/19	T	64.7	NR	F	791.54	31.37	2.20	154.7	5.47	119.7	1.07	1.23	5.44	26	10.7
392	5/13/19	T	63	NR	F	183.94	6.04	1.98	152.2	4.19	116.7	0.93	1.18	4.00	11.6	9.6
402	5/14/19	T	59.3	NR	U	357.86	17.63	2.28	150.5	4.62	118.0	1.04	1.28	3.72	19.5	13.2
419	5/16/19	T	59.8	NR	U	126.41	5.65	2.33	155.5	4.66	119.4	1.06	1.42	3.94	19.4	10.0
431	5/16/19	T	57.9	NR	U	204.46	7.80	2.24	153.0	4.68	120.7	1.02	1.65	4.61	19.5	12.9
1330	6/8/16	T	58.7	30	U	1024.24	39.57	3.65	153.2	6.39	117.5	1.15	1.21	6.61	22.8	22.5
1331	6/9/16	T	59.3	26.5	U	1050.64	53.63	1.91	150.6	5.38	119.9	1.01	1.37	3.78	19.7	18.2
1423	5/18/16	T	63.7	NR	M	438.61	16.19	1.24	148.9	4.94	119.9	0.72	1.06	4.00	15.9	16.8
1424	5/19/16	T	56.6	22.5	U	764.45	37.36	2.13	150.9	6.15	116.0	1.03	1.32	4.78	25.2	40.4
1425	5/19/16	T	63.6	NR	F	600.39	18.39	1.17	147.7	4.75	116.0	0.89	1.08	4.56	22.8	26.1
1426	5/21/16	T	61.3	NR	M	891.37	33.42	1.61	148.9	5.23	117.5	1.09	1.16	4.06	20.7	28.6
1427	5/21/16	T	67.5	NR	F	426.60	25.65	2.05	151.2	5.00	116.6	0.99	1.07	5.22	22.5	33.9
1428	5/22/16	T	61.5	NR	F	585.50	21.35	2.08	152.2	5.23	119.6	0.90	1.38	5.67	19.1	34.6
1429	5/23/16	T	66.1	NR	M	497.15	36.91	1.33	154.5	5.39	118.7	0.97	0.93	4.67	24.6	26.1
1430	6/22/16	T	64.4	33	F	777.75	31.50	1.20	QNS	QNS	QNS	QNS	QNS	QNS	QNS	QNS
